# An Extra Power Saving Scheme for Prolonging Lifetime of Mobile Handset in the 4G Mobile Networks

**DOI:** 10.1371/journal.pone.0103429

**Published:** 2014-08-04

**Authors:** Jenhui Chen, Woei-Hwa Tarn, Jiann-Der Lee

**Affiliations:** 1 Department of Computer Science and Information Engineering, School of Electrical and Computer Engineering, College of Engineering, Chang Gung University, Kweishan, Taoyuan, Taiwan; 2 Department of Electrical Engineering, School of Electrical and Computer Engineering, College of Engineering, Chang Gung University, Kweishan, Taoyuan, Taiwan; Xiamen University, China

## Abstract

In the fourth generation or next generation networks, services of non-real-time variable bit rate (NRT-VBR) and best effort (BE) will dominate over 85% of the total traffic in the networks. In this paper, we study the power saving mechanism of NRT-VBR and BE services for mobile handsets (MHs) to prolong their battery lifetime (i.e., the sustained operation duration) in the fourth generation networks. Because the priority of NRT-VBR and BE is lower than that of real-time VBR (RT-VBR) or guaranteed bit rate (GBR) services, we investigate an extended sleep mode for lower priority services (e.g., NRT-VBR and BE) in an MH to conserve the energy. The extended sleep mode is used when the MH wakes up from the sleep mode but it cannot obtain the bandwidth from base station (BS). The proposed mechanism, named *extra power saving scheme* (EPSS), uses the 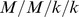 Markovian queuing model to estimate the extended sleep duration to let MHs conserve their battery energy when the networks traffic is congested. To study the performance of EPSS, an accurate analysis model of energy is presented and validated by taking a series of simulations. Numerical experiments show that EPSS can achieve 43% extra energy conservation at most when downlink resource is saturated. We conclude that the energy of MHs can be conserved further by applying EPSS when the traffic load is saturated. The effect of energy saving becomes more obvious when the portion of NRT-VBR and BE services is greater than that of RT-VBR and GBR services.

## Introduction

The fourth generation (4G) personal communication networks provide telecommunication and data access together for nomadic users by integrating telephone and computer networks into a single system [1], [2]. These nomadic users access Internet services via mobile handsets (MHs), e.g., smart phones or pads, and these MHs work relying on the battery energy. According to the latest reports of statistics of mobile data traffic [3], [4], the non-real-time variable bit rate (NRT-VBR) and best effort (BE) traffic, e.g., data (Internet downloading, web browsing, E-mail, and social networking), file sharing (FTP and P2P), and video (buffered video streaming), dominates around 86.4% of mobile data traffic in early 2013. These reports forecast that mobile data traffic will grow at a compound annual growth rate of 66% from 2012 to 2017, reaching 11.2 exabytes per month by 2017 [3]; video streaming will dominate two-thirds of the traffic, where NRT video (buffered video streaming) dominates three-fourths of the video streaming traffic. However, according to the guideline of related 4G standards [5], [6], NRT-VBR service does not have the high priority to acquire the system resource (e.g., bandwidth) for transmission [7]. It means that less system resources are allocated to high proportion of services. This certainly leads a serious competition [8].

On the other hand, because the energy of the MH battery is limited, how to prolong the lifetime of the MH battery to support multiple services has become an valuable issue. Many power saving mechanisms (PSMs) have been broadly studied such as adaptive traffic coalescing (ATC) scheme [9], power saving studies based on quality-of-service (QoS) constraints [10], statistical sleep window control (SSWC) approach [11], fold-and-demultiplex (FD) method for power saving class (PSC) type I and II services [12], and a hybrid mechanism for voice over IP (VoIP) service to improve power saving efficiency [13]. Because PSM uses the method that an MH turns off its radio transceiver in idle time to conserve its energy, too much waiting time will decrease the efficiency of PSM. Those mentioned PSMs, however, do not consider a problem that MHs may not obtain the downlink bandwidth and thus waste their energy due to traffic congestion. Actually, the problem of wasting battery energy on waiting for available bandwidth can be solved by implementing a smarter scheduler in a base station (BS) to order those MHs to enter a pre-calculated *extended sleep duration* for energy conservation. In this paper, we present an MH PSM named *extra power saving scheme* (EPSS) to prolong the lifetime of the MH battery by avoiding those unnecessary and uncertain waiting time for available bandwidth. From the performance evaluation, we find that the proposed mechanism can achieve better power saving efficiency as compared with the 4G communication protocols when the traffic load is large.

The rest of this paper are organized as follows. A detailed description of the unnecessary energy wasting because MHs wait for available bandwidth in 4G systems and the solution of EPSS is given in Section. The analytical model of the proposed EPSS is presented to show how to obtain the estimated extended time and derive the energy consumption in Section. Section shows the validation of the analytical model and related numerical experiments. Finally, the conclusion is given in Section.

## Extra Power Saving Scheme

### Problem Description

Latest 4G communication techniques, the IEEE 802.16m [5] and long-term evolution advanced (LTE-A) [6], utilize 100% packet-switched air interface to provide numerous services for MHs. To ensure that the data receiving meet the QoS requirement of variant application services, these two standards provide different QoS classes for different application services which are treated as service flows. Table 1 and Table 2 summarize the priority order of QoS classes of IEEE 802.16m and LTE-A, respectively. As mentioned in the preceding section, around 86.4% of Internet traffic is NRT-VBR, BE, and non-GBR types. These types of traffic are lower priority services as compared with GBR services [4]. Because the NRT-VBR services have the delay-tolerant property [15], they are usually served in a lower priority as compared with real-time (RT) and guaranteed bit rate (GBR) services. This setting will lead to the consequence that NRT-VBR and non-GBR services cannot obtain the bandwidth when they compete with services having higher priority than theirs. This problem will also appear when all services are NRT-VBR or non-GBR and they compete bandwidth with each other because these services are served in a first come, first served (FCFS) basis. Then we can conclude if the downlink packet has higher QoS priority, e.g., rtPS/GBR service, the scheduler will allocate the resources first to support its transmission. In contrast, if the downlink packages are nrtPS/non-GBR services, it may need longer waiting time till the scheduler serves the services with higher QoS priority. Especially when the system is in saturated condition [16].

**Table 1:** IEEE 802.16 QoS Classes and Priorities [14].

service	abbrev.	priority	definition	typical applications
Unsolicited Grant Service	UGS	1	Real-time data streams comprising fixed-size data packets issued at periodic intervals	T1/E1 transport
Extended Real-time Polling Service	ertPS	2	Real-time service flows that generate variable-sized data packets on a periodic basis	VoIP
Real-time Polling Service	rtPS	3	Real-time data streams comprising variable-sized data packets that are issued at periodic intervals	MPEG Video
Non-real-time Polling Service	nrtPS	4	Delay-tolerant data streams comprising variable-sized data packets for which a minimum data rate is required	FTP with guaranteed minimum throughput
Best Effort	BE	5	Data streams for which no minimum service level is required and therefore may be handled on a space-available basis	HTTP

**Table 2:** 3GPP Standardized QCI and Priorities [17].

QCI	Priority	Resource Type	Delay Budget	Example Services
1	2	GBR	100 ms	Conversational Voice
2	4	GBR	150 ms	Conversational Video (Live Streaming)
3	3	GBR	50 ms	Real-Time Gaming
4	5	GBR	300 ms	Non-Conversational Video (Buffered Streaming)
5	1	non-GBR	100 ms	IMS Signaling
6	6	non-GBR	300 ms	Video (Buffered Streaming), TCP-based (e.g. www, e-mail, chat, ftp, p2p file sharing, progressive video, etc.)
7	7	non-GBR	100 ms	Voice, Video, Interactive Gaming
8	8	non-GBR	300 ms	Video (buffered streaming) TCP-based (e.g., www, email, chat, FTP P2P file sharing, progressive video, etc.)
9	9	non-GBR	300 ms	Video (buffered streaming) TCP-based (e.g., www, email, chat, FTP P2P file sharing, progressive video, etc.)

In order to prolong the battery lifetime of an MH, the PSM turns off an MH radio interface when the MH is in idle duration. The IEEE 802.16m PSM provides three types of PSCs for different service types to conserve the battery energy. The PSC type I is used for NRT and BE traffic, PSC type II is used for unsolicited grant service (UGS) and RT-VBR traffic, and PSC type III is used for multicast and management traffic, respectively. Similarly, the LTE-A PSM uses discontinuous reception and discontinuous transmission (DRX/DTX) mechanism to conserve the battery energy in downlink and uplink connections, respectively. When an MH is in active mode, it can receive the packets from the BS regularly. When an MH is in sleep mode, it turns off the transceiver power and wakes up periodically in a period of time (i.e., listening window). In the listening window, the MH listens the message from the BS to confirm whether the BS has any packets to the MH. If the BS has any packets to the MH, it must inform the MH in the listening window to let the MH keep awake and receive the packet. If the MH does not receive a positive notification from the BS, it will go back to another sleep cycle.

Based on preceding described PSM, when the MH is in sleep mode, the BS cannot communicate with the MH except in the pre-negotiated listening window. If the BS has packets to the MH, the BS has to send a positive notification to inform the MH. Because the positive notification does not include the information of the bandwidth allocation message, the MH must keep awake until it receives the downlink bandwidth allocation information. This leads the MH to take more unnecessary energy consumption to wait, especially the scheduler cannot allocate available bandwidth for the nrtPS/non-GBR transmission. We find that both of IEEE 802.16 and LTE-A do not have effective solution for this problem. According to the aforementioned increasing trend of nrtPS/non-GBR services in Section, this problem will become more series in the future.

### Mechanism

As mentioned in Section and Subsection, when an MH receives a positive notification in the listening window, the MH keeps awake to wait for the downlink bandwidth allocation message to receive upcoming packets. To alleviate the energy wasting of MHs in case the downlink bandwidth are not available, EPSS modifies the positive notification message (e.g., the AAI_TRF-IND message used in IEEE 802.16m [5] and the physical downlink control channel (PDCCH) used in LTE-A [6]) to let the BS be able to notify those MHs which cannot obtain the bandwidth immediately to enter the extended sleep mode for battery power conservation. We note that EPSS can be applied to either IEEE 802.16m or LTE-A protocol because both of these two standards use the similar mechanism to perform the PSM for nrtPS or non-GBR connections and have the same problem of energy wasting.

**Figure 1:**
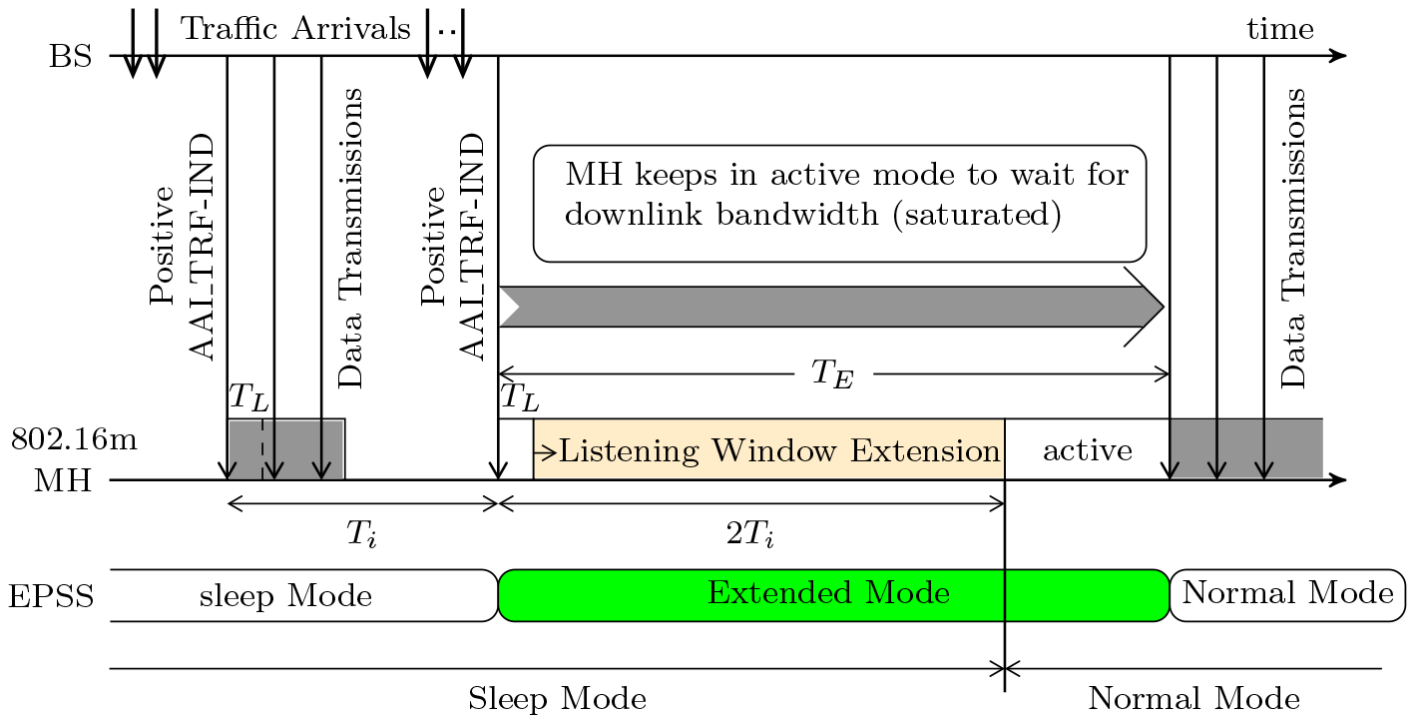
The type I power saving operation in IEEE 802.16m standards.

[Figure 1] illustrates the operation of PSC type I in the IEEE 802.16m protocol and the described solution of EPSS. When an MH enters the sleep mode, the MH repeats the sleep cycle with binary exponential increment, i.e., 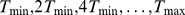. A listening window 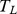 is at the beginning of each sleep cycle (except the first one) to let MHs receive AAI_TRF-IND signaling messages. If there is any downlink packets destined to the MH, BS will use SLPID field and bitmap in AAI_TRF-IND to notify the MH and transmit these packets later. When the MH receive the notification in the listening window, the MH will keep awake to wait the DL-MAP and receive the packets. If the packet transmission time is longer than the default listening window, the BS sends a sleep control header (SCH) to extend the listening window (e.g., the grey interval in 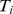) till the end of current sleep cycle. When the MH finishes receiving the downlink traffic, i.e., there is no packets in the buffer of the BS for the MH, the BS will send an SCH to terminate the downlink transmission and the MH will go back to the sleep window till the end of sleep cycle. As was mentioned above, current IEEE 802.16m standard only assume an ideal condition that the MH will obtain the downlink bandwidth immediately after receiving the positive AAI_TRF-IND message. Nevertheless, this is not true that the BS can provide needed bandwidth to the MH in time to receive data. As we described in Section and Subsection, nrtPS/non-GBR services have lower QoS priority for bandwidth allocation than rtPS/GBR, this leads the MH to wait longer for available bandwidth. The worst case may happen when the bandwidth is saturated that the MH keep awake till the end of the sleep cycle even longer (e.g., the grey arrow in 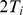). Then, the detail of extra power saving scheme is described as follows.

**Figure 2:**
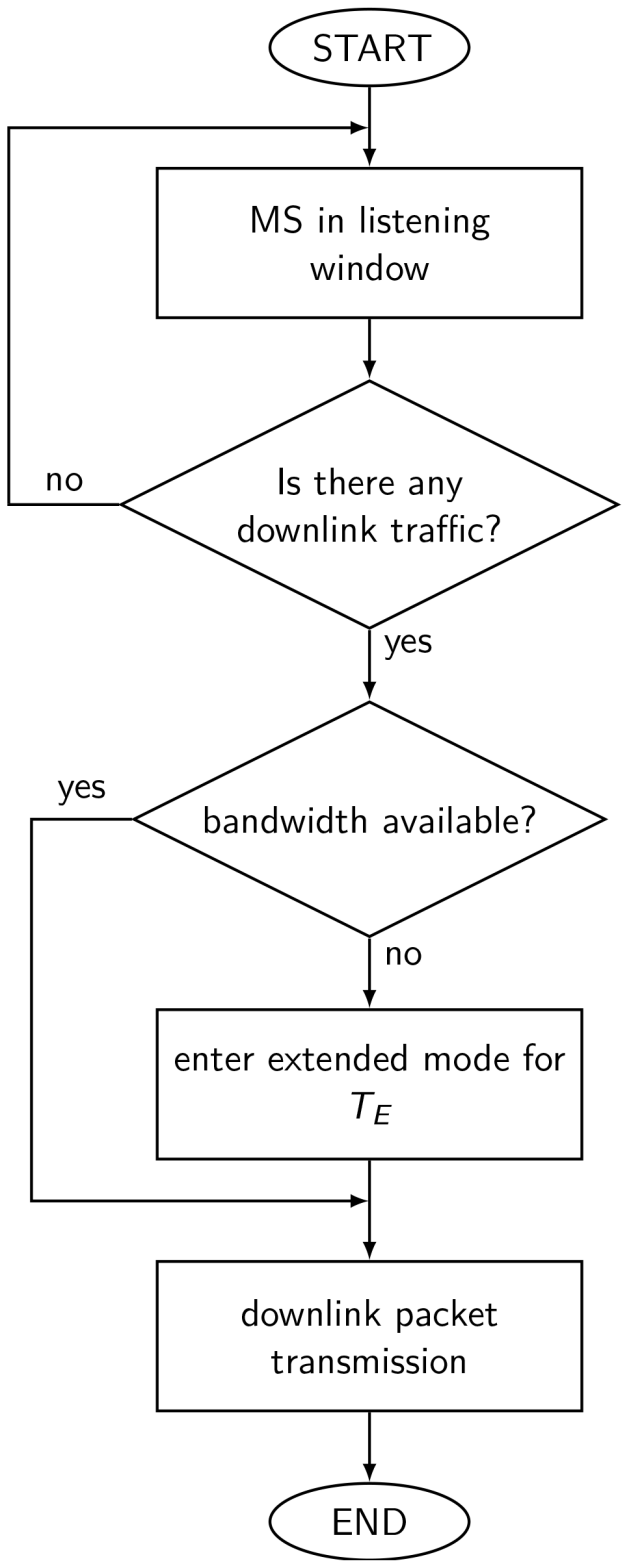
The algorithm of EPSS scheme.

If the BS use AAI_TRF-IND message to keep the MH awake but the scheduler cannot provide available bandwidth, the BS will send the AAI_SLP-RSP messages to update the sleep parameter of MH. Then the MH will enter an extended mode for a specified time duration 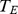. 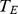 is estimated from the traffic and available bandwidth at that time. In the extended mode, the MH can turn off its transceiver as it is in sleep mode and avoid the energy wasting. The algorithm of EPSS is shown in [Figure 2]. When the MH in the listening window of sleep mode, the BS checks if there is any downlink traffic for it. If there is no traffic for the MH, it will go back to next sleep window. Otherwise, the BS will check whether the bandwidth is available. If the bandwidth is available, then the packet transmission will be performed. On the contrary, the MH will be ordered to enter the extended mode for 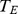. When the MH sleeps for 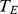, it will directly perform the packet transmission and the scheduler should promote the MH to higher priority. In this way, the MH can decrease the unnecessary waiting time when the system is saturated and achieve load balance of the system.

## System Model

The system is modeled as a one BS to 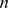 MHs communication model. Because the PSM is used for downlink transmission, only downlink bandwidth is considered. The service queue of downlink for each MH follows the FCFS basis. The BS only maintains 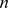 NRT-VBR downlink queue for 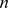 MHs (i.e., each MH has only one downlink queue for NRT and BE services in the BS buffer). For simplicity, a simple bandwidth allocation policy is considered. Since the downlink bandwidth resource is limited, we assume that 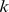 resource spaces in total at most are allowed to assign among *downlink sessions* in a frame. In this paper, all NRT-VBR/non-GBR/BE services accepted by the call admission control (CAC) mechanism are treated as sessions. In order to increase bandwidth utilization, the number of allowed sessions could be greater than the number of total resource spaces per frame, i.e., 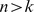.

**Figure 3:**
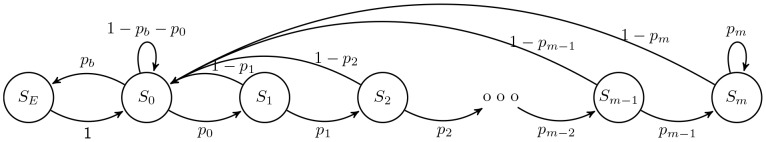
The state transition diagram of EPSS.



The PSM of EPSS is modeled as the Markov chain model [18] as shown in [Figure 3]. Similar models and assumptions can be found in [19], [20], however, it is totally different from EPSS model and has to be redesigned. When an MH enters the sleep mode, the MH repeats the sleep cycle 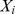, where

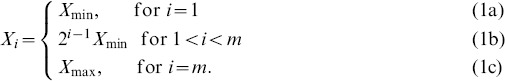



An MH wakes up periodically to listen AAI_TRF-IND during 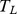 at the beginning of each sleep cycle 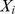, where 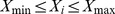, and then goes back to sleep window till the end of the sleep cycle. The value of sleep window 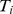 in different states is 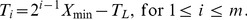
(2)


In the state transition diagram, all the states are represented as a set 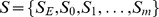, where 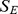 represents the state that the MH is informed with a positive AAI_TRF-IND but cannot be provided resource. In EPSS, the MH will enter the extended sleep mode to avoid unnecessary energy consumption. 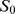 represents the normal mode state and the subset 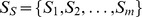 represents the sleep mode states. All elements in 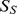 follow the sequence of 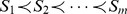 in chronological order. An MH sleeps a time duration 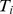 in the state 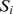. After the duration time 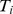 (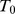 represents the idle time before MH enters the sleep mode and 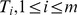, represents the 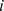
th sleep window), an MH may transit from 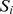 to 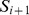 with the probability 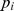 or enter the normal state 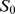 with the probability 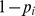 depending on the downlink traffic to the MH. Assume the downlink arrivals to an MH are a Poisson process with a mean rate 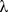 [21], [22]. Let 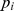 be the probability that the MH transits from 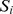 to 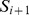, where 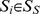, in the sleep mode. Because the probability 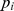 depends on the probability of no downlink arrivals to the MH during a time interval 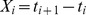, where 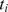 represents the time the MH enters 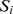, we have 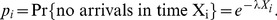
(3)


### State Transition Probability of EPSS

In EPSS, the MH will enter 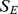 if it cannot obtain the resource immediately, i.e., the network load is saturated. Let 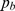 be the downlink transmission blocking probability (i.e., the transition probability from 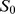 to 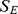 as shown in [Figure 3]). As mentioned in Section, the system has 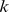 parallel sessions in a frame and 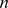 sessions are permitted to be served in the system. The queueing model of EPSS can be formulated as the *Erlang-B formula* [18] with an 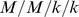 model. According to the 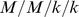 model, 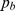 can be obtained by 
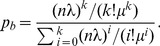
(4)


Since the transition probability from 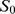 to 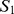 is 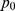 (see [Figure 3]), the state transition probability from 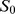 to 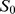 (i.e., an MH stays in 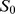) is equal to 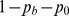. In EPSS, since an MH transits from extended mode to normal mode (i.e., 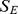 to 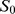) without any conditions once the extended time is decreased to zero, the state transition probability from 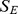 to 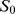 is equal to 1. According to the *flow balance* property of steady state in Markov chain of EPSS, we have 
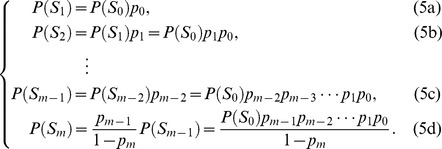



The sleep mode probability can be obtained by summing up all probabilities of sleep mode states, then according to (5), we can obtain 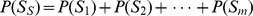


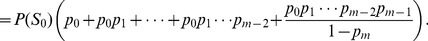
(6)


Following the steady state property, in state 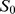, we have 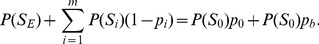
(7)


Solving 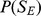 for (7), we have 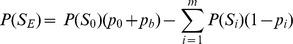


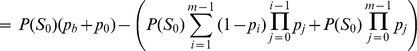


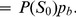
(8)


To solve 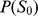, we use the axiom of probability theory 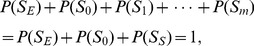
(9)


Substituting (6) and (8), into (9) leads to 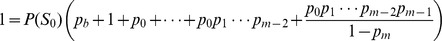


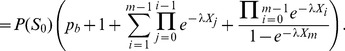
(10)


Then 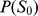 can be obtain as 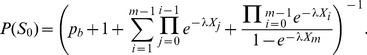
(11)


Substitute (11) into (8) and (5), we can obtain the steady state probability 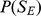 and 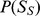.

### Energy Consumption

The energy consumption can be derived by using preceding obtained probabilities 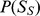, 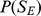, and 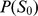. Let 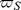 and 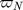 denote the energy consumption per unit time in sleep mode and normal mode, respectively. Thus, the energy consumption of the EPSS per unit time, denoted as 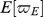, can be obtained by 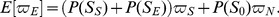
(12)


In comparison with EPSS, we use the similar model and obtain the steady state probability of the legacy IEEE 802.16 PSM 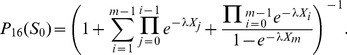
(13)


In a similar way, we can substitute the probability 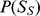 and derive the energy consumption of the legacy IEEE 802.16 PSM per unit time, denoted as 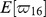, as 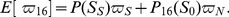
(14)


### Extended Time Estimation

**Figure 4.The:**
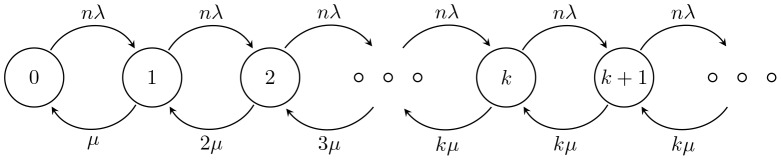
rate transition diagram for the 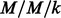 queueing mode.

The delay of access network bandwidth is an important metric of performance. In order to estimate 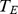, we take the 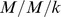 Markovian queueing model to derive the estimation function of 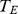. We assume that there are at most 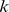 parallel sessions (transmissions) allowed at a frame in the system. [Figure 4] shows the Markov chain model with states 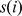, where 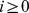 represents the number of busy channels. When the process is in state 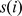, for 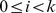, 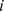 channels are busy. The effective downlink traffic to an BS at 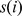 is 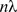, where 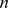 represents the number of MH served by the BS. Suppose the downlink transmission time is exponentially distributed with mean 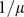. Because a busy channel is released with rate 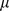 (i.e., the service rate of a channel), the process moves from 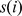 to 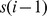 for 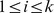 with rate 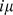.

According to the process, the steady-state probabilities 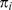 for 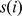 can be expressed as 
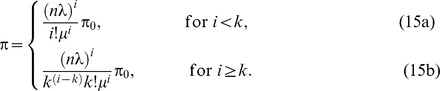



To solve 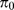, we use 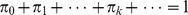 to obtain 
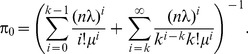
(16)


Based on the derivation of [18], we can rewrite (16) by substituting 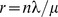 and 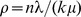 as 
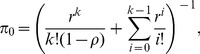
(17)where 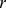 represents the work load rate to a server (i.e., a channel) and 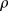 means the work load rate to the system. We notice that the reason we use 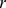 and 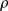 to replace 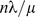 and 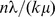 here is to easily understand the status of the traffic load in the whole system.

Let 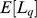 be the expected number of MHs that cannot be served and enters 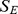, i.e., 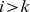. Then 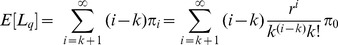


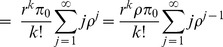


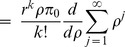


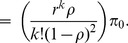
(18)


Substituting (17) into (18), we get 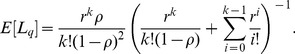
(19)


Because the downlink transmission arrival rate of nrt-PS to the BS is 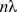, it is easy to use Little's formula to get 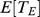: 
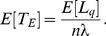
(20)


From (20), the BS can estimate the expected time duration 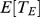 according to the current traffic load to the MH. Note that the computed 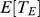 will be adopted by the scheduler of the BS to schedule the needed resource to the MH accordingly after the period of sleeping time 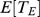. Thus, the MH goes to 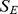 for 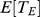 and comes back to receive the downlink traffic. Let 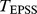 denote the total time an MH wakes from the sleep mode and finishes the downlink transmission. Thus the mean downlink transmission delay of each downlink attempt achieved by EPSS will be 
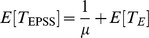


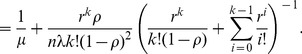
(21)


## Numerical Experiments

### Simulation Model

The simulator is developed by the authors to evaluate the energy consumption of the legacy IEEE 802.16 PSM and EPSS. The simulation model is based on all system assumptions presented in Section. There are 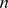 independent MHs which connect with one BS for Internet access. The downlink traffic to an MH is an independent Poisson source with an aggregate mean burst data generation rate of 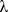 packet arrivals/frame and the total traffic to the downlink channel is equal to 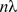. The arrival data consist of consecutive packets arrive at the BS and are scheduled for downlink burst transmission. Once the downlink traffic arrives at the BS, the BS will wake up the MH at the next listening window if the MH is in the sleep mode. The scheduler follows the FCFS basis to schedule the arrival data. In EPSS, we emulate the behavior of MHs which are served. If an MH does not get enough resource to receive the downlink traffic after it wakes up from sleep mode. The BS will count 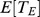 that is obtained from (20) and order the MH to enter the extended mode. After the duration of 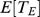, the MH will wake up from the extended mode and get a priority queue to be allocated the DL radio resource.

The transmission of arrival in a session is assumed exponentially distributed with mean rate 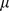 packet arrivals/frame. Thus the total downlink channel capacity can be represented as 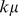, where 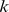 indicates the scheduled number of sessions per frame. When 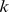 increases, this implies the number of served session increases, then the allocation resource for each 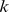 will decrease. When 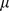 increases, this implies the modulation rate (carried bits per one logic resource unit) increases. In other words, the larger the 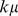 is, the larger the capacity of service in a frame is. In the simulation, the MH will enter the normal mode after it goes out the extended mode and completes the reception of the downlink data. The MH will enter the sleep mode again till it successfully waits a 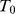 period without any downlink traffic. The provided work load for the system is set as 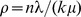.

To study the effect of the power saving achieved by EPSS, we investigate the relationship between 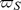 and 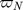. Let 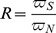
(22)indicate the ratio of energy consumption in the sleep mode to the normal mode. To normalize the energy consumption we let 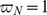. For example, when 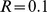, which means the energy consumption in the sleep mode is 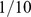 of the energy consumption in the normal mode, i.e., 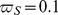. In the following evaluation and comparison, the normalized energy consumption per frame, denoted by 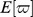, is used as our performance measurement. When 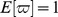, it implies that the MHs are always in normal mode and do not take advantage of sleep mode.

**Figure 5:**
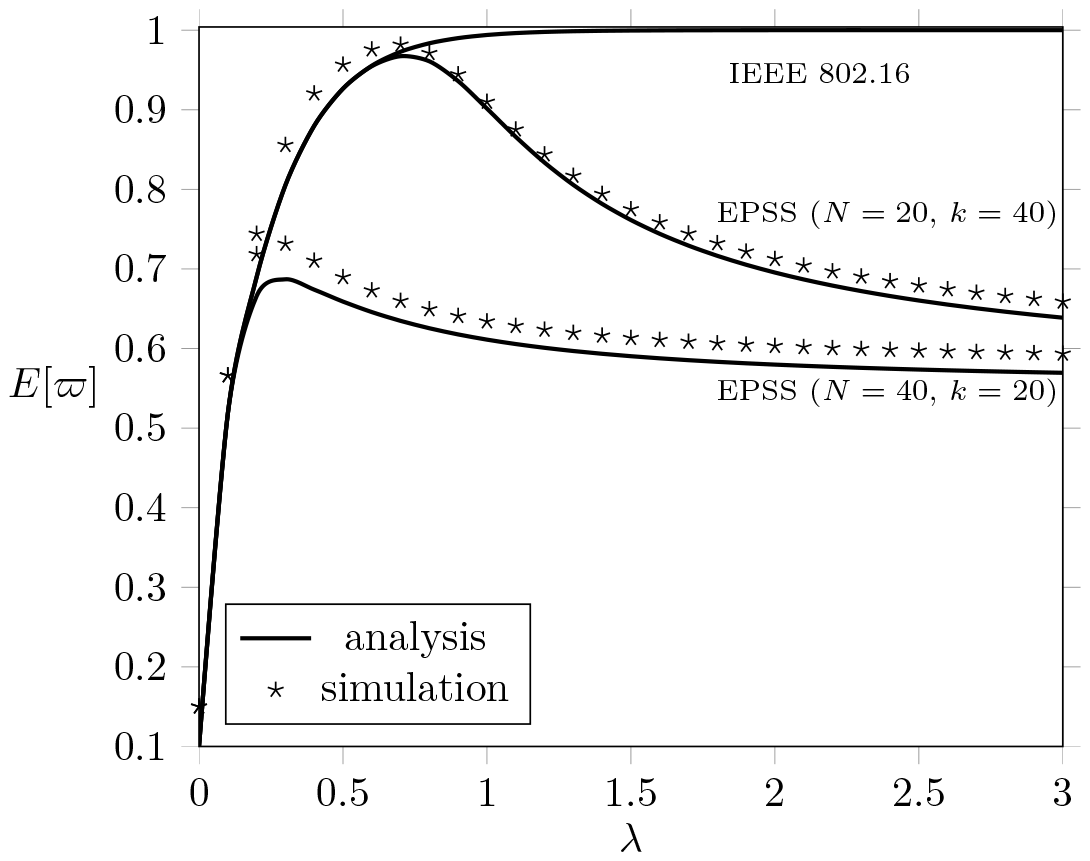
Simulation versus analysis in terms of arrival rate 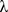 when 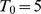, 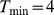, 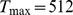, and 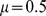.

Other system parameters are set as follows: the initial sleep window 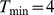, the listening window 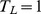, the number of states in sleep mode 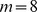, i.e., 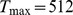, the given idle time duration 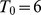 to enter the sleep mode, the ratio of energy consumption 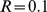, and the channel capacity 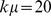. Our analytical model has been validated against the simulation experiments. We perform the simulation 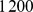 times to average each plotting value as shown in [Figure 5]. The solid lines are the numerical results obtained from our derived formulae. We can see from [Figure 5] that the simulation results matches the numerical results.

### Numerical Examples

In this subsection, we use some numerical examples to illustrate the effects of the number of MHs 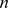, the initial idle time duration 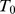, the downlink channel capacity 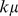, the maximum number of sleep mode states 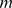, the energy consumption ratio 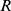, and 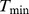 on output measure 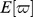 (the normalized energy consumption). Further, we also show the effect of 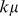 on the downlink transmission delay 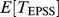 caused by EPSS.

#### Effect of 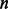


**Figure 6:**
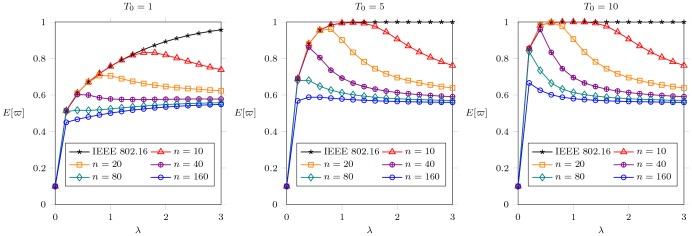
The effect of different 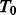 on 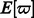 when 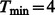, 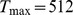, 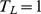, 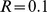, 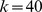, and 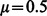.

[Figure 6] illustrates the output measure 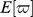 by varying different downlink arrival rate 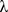 when 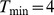, 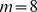, 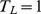, 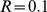, and 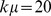 (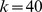, 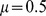) under different 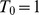, 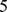, and 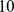. [Figure 6] shows that 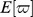 of IEEE 802.16 PSM increases as 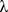 increases (the larger the downlink arrival rate is, the higher the energy consumption it will be). Taking the subfigure 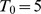 as an example, the upper bound of IEEE 802.16 reaches 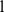 as 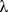 reaches 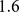 (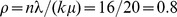). This result implies that the energy consumption achieved by IEEE 802.16 cannot be reduced any more when the work load is approximately saturated.

In contrast, initially 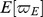 increases as 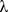 increases but 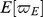 will decreases when download traffic load approaches saturation, i.e., 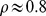. Unlike the IEEE 802.16 PSM, EPSS estimates an extended sleep time to let MH enter the extended mode for extra power saving, thus the energy consumption will decrease accordingly when 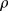 is more than 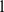. [Figure 6] shows that EPSS can achieve 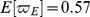 at most when 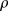 is very large. This implies that EPSS is capable of conserving the energy consumption as the channel utilization is saturated.

[Figure 6] shows that the energy consumption is proportionally decreasing when 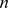 increases. We first note that 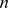 is a major factor that determines 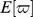. The larger the number of 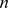 implies the more competition among MHs to obtain the bandwidth is intense. In other words, MHs in highly competitive circumstance, the delay of obtaining the bandwidth for transmission is longer. And thus EPSS enforces MHs to enter the extended mode, which could avoid the MH spending unnecessary idle time to waste the energy.

#### Effect of 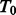


[Figure 6] additionally plots 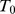 against 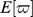, where 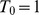, 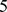, and 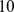 (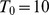 is a maximum recommended value for practice [23]). This consequence shows the value of 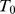 will affect 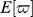. When 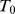 is larger, MHs will obtain lower energy saving efficiency, i.e., 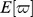 is higher, because an MH should wait a longer time to enter the sleep mode. It makes sense that MHs get quicker response time to receive the downlink transmission if the MH prolongs 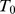 to enter the sleep mode. However, this effect will decrease the efficiency of energy conservation.

#### Effect of 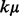


**Figure 7:**
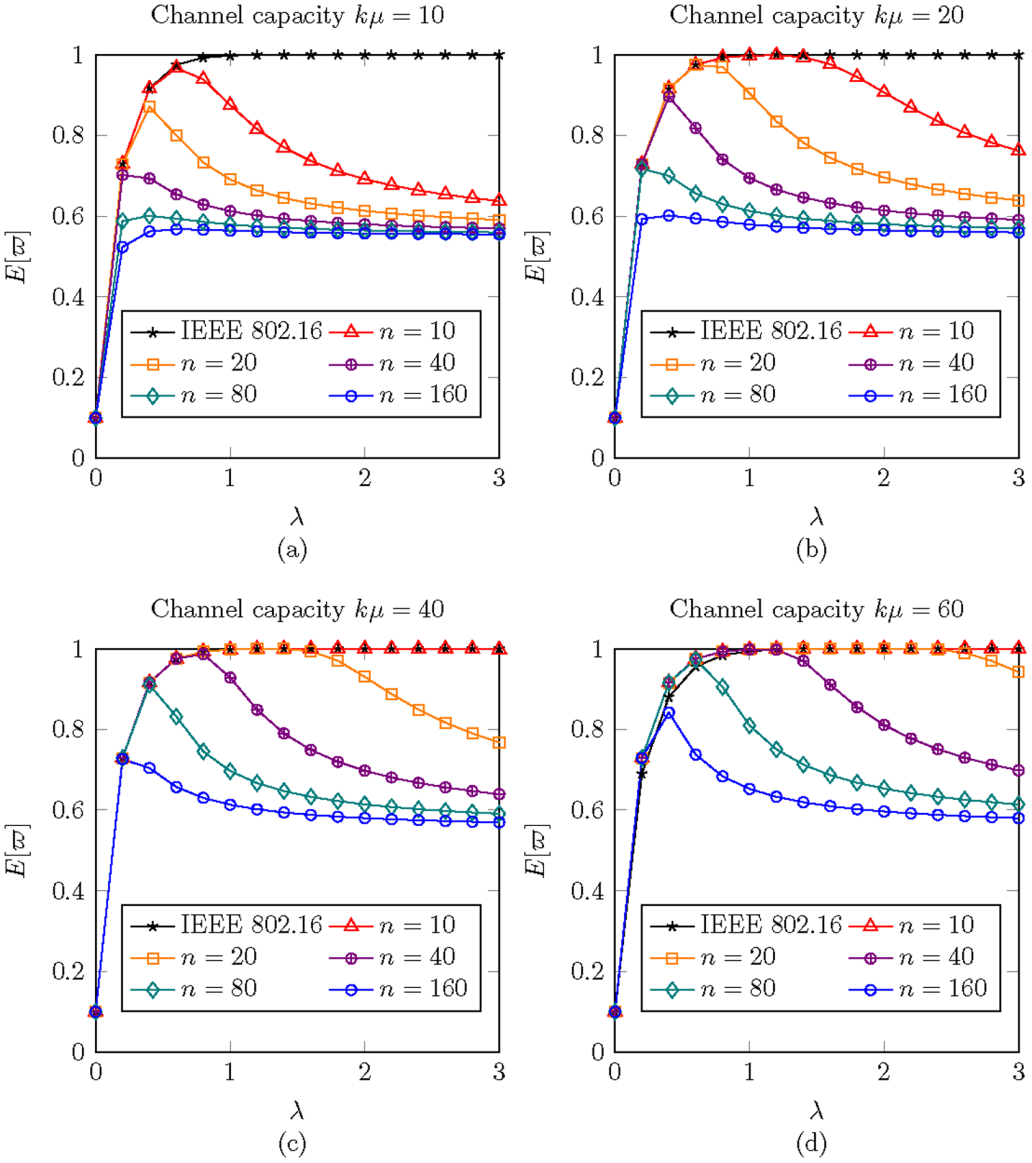
The effect of different 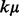 on 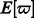 when 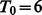, 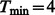, 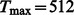, 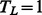, 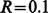, and 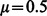.

[Figure 7] plots 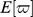 against the arrival rate 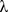 to each MH. Each subfigure represents different 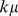 as 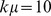, 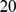, 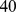, and 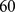, where 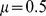, i.e., 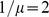 frames. The parameter 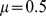 implies each arrival to an MH dominates 2 frames long in average to be served. [Figure 7] shows that, in general, 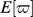 grows up proportionally with the increase of 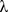 before the offered load rate is saturated (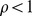). First, 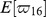 will reach 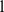 when 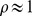. The energy consumption cannot be reduced further because the IEEE 802.16 PSM cannot reflect the offered load. On the contrary, EPSS can reflect the traffic load and enforce the MHs to enter the extended mode for power conservation.

[Figure 7] shows that energy consumption is proportional to 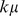. For example, in [Figure 7](a), EPSS will conserve more energy when the channel capacity 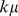 is small. In contrast to [Figure 7](c), [Figure 7](d) shows the 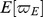 is the same as 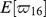 when 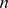 is small and 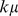 is large. This result indicates that EPSS exhibits its effect on 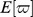 as the network is saturated. There are two cases to lead the situation of network saturation. One is 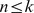, i.e., the number of MHs 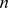 less than or equal to the number of serving channel in a frame. The other one is 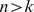, i.e., the number of MHs 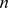 greater than the number of serving channel in a frame. In case one, the system saturation only takes place when each MH has so much traffic to be received, so it is always in normal mode. In the second case, the resource is less than the request of all MHs. In this case, it leads some MHs idle to wait the BS to allocate resource. It motivates the EPSS to be proposed to reduce the MH idle time and conserve more energy. The effect of EPSS on 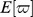 will be more obvious when 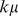 is small or 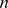 is large.

**Figure 8:**
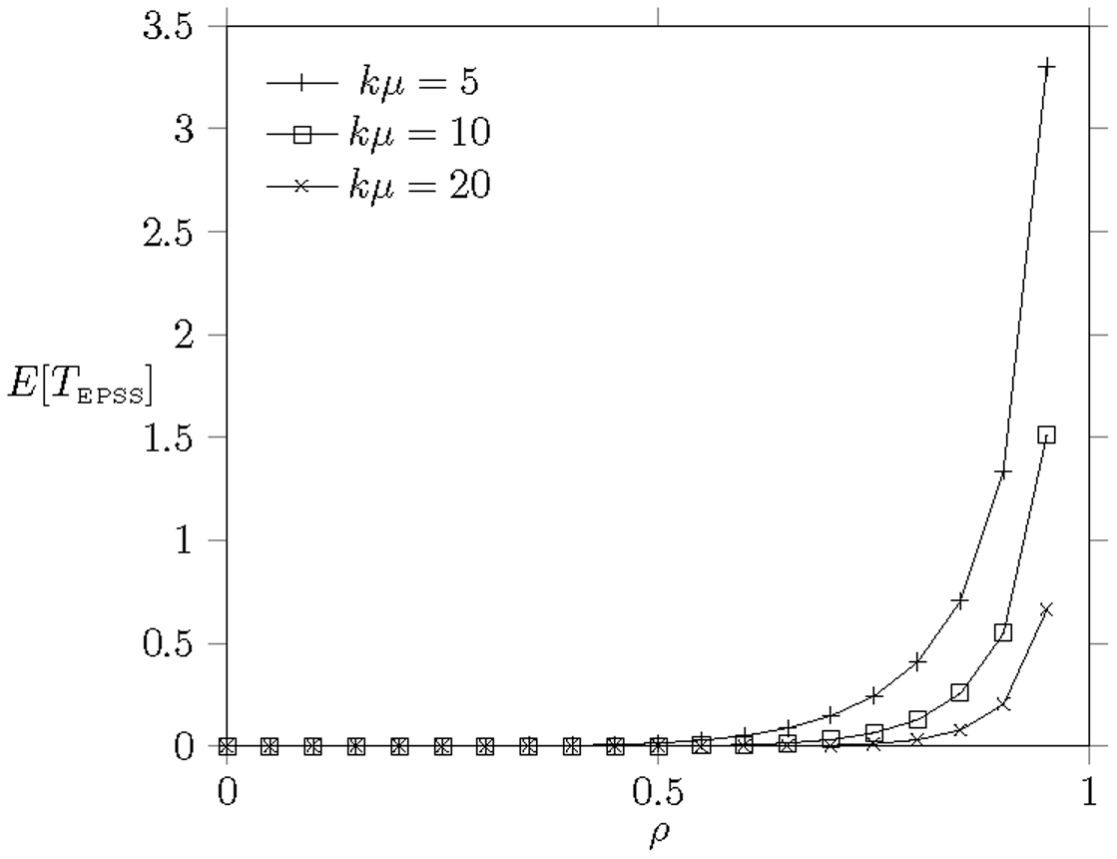
The downlink 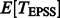 versus work load 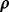 when 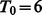, 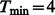, 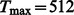, 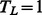, 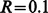, 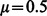, and 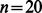.

Another affected result, the mean downlink transmission delay 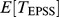, caused by 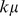 is shown in [Figure 8]. As we can expect that 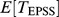 grows rapidly as the network load is saturated, i.e., 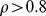, because there are no more bandwidth to be allocated. This result explains the phenomenon that 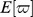 decreases when 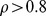 (see [Figure 7]) because EPSS enforces the MHs into the extended mode for energy conservation.

#### Effect of 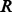


**Figure 9:**
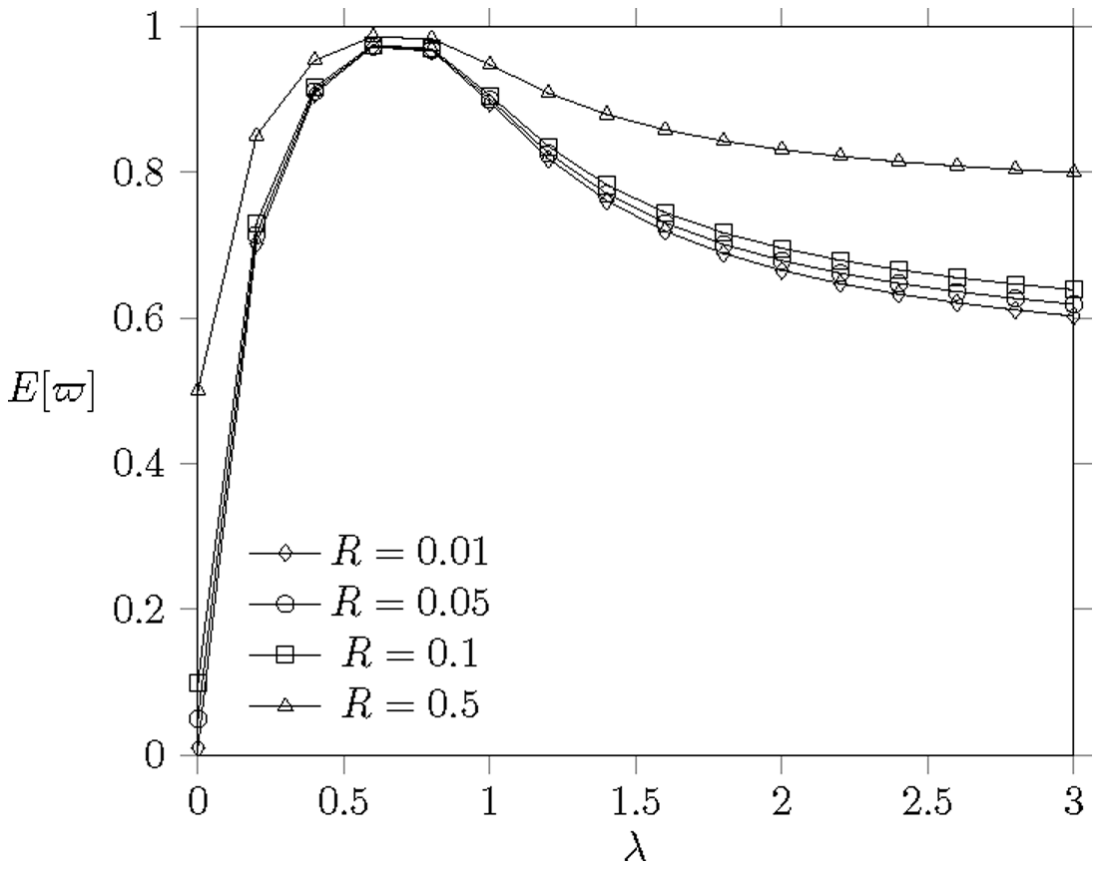
The energy consumption under different 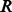 when 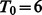, 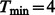, 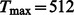, 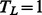, 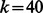, 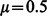, and 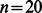.

[Figure 9] illustrates the effect of energy consumption of sleep mode to normal mode on 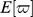. The revealed data indicate that the higher ratio of 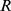 achieves lower 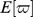. The improvement is significant when 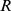 from 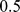 to 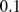, i.e., 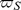 is from 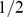 of 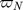 to 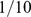 of 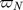. However, when 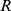 from 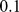 to 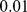 (10 time smaller than 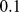), the improvement of 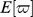 is getting smaller. The gap of 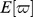 improvement between the gap between 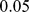 and 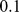 and the gap between 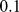 and 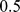 is 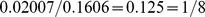. This observation means the improvement from 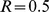 to 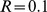 is 8 times of the improvement from 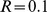 to 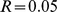. Based on the discovery, we recommend the design of 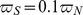 is an efficient value to achieve good energy conservation.

### Realistic Traffic

In the following, we present a realistic traffic study of buffered video streaming and web browsing to investigate the effect of EPSS on energy conservation. The traffic model refers to the statistical results in [3], [4]. We have developed a C++ program to emulate the two traffic types. The system parameters used in the simulation follow the IEEE 802.16 standard and are shown in Table 3. The minimum resource allocation size is 12 logic resource units (LRUs) where each LRU can bear 432 bits. Therefore, there are 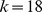 available resource blocks a frame (downlink) deducting the control overheads, e.g., super frame headers, mapping information, and control messages. We assume the ratio of video streaming traffic to web browsing traffic is 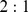. Each MH can only make a request to the BS for service. The simulation time is 10 minutes (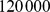 frames) long to ensure that simulation results are stable.

**Table 3:** Analysis Parameters for IEEE 802.16.

Parameter	Value
simulation time	120 000 frames
bandwidth	56 Mbps
frame size	5 ms
downlink/uplink ratio per frame	5/3
LRU size (16-QAM, coding rate 3/4)	432 bits
minimum resource allocation size per frame	12 LRUs
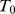	5 frames
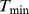	4 frames
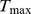	512 frames
mean size of video streaming per request	176 Mbits
mean size of web browsing per request	6 Mbits

The buffered video streaming traffic we consider here is the behavior of people watching the YouTube video clip. The resolution of video clip is 480p (854 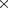 480 pixels) resolution, which consumes bandwidth about 1000 kbps. The length of video clip is 3 minutes long on average, i.e., each downloading of a video clip will consume up to 176 Mbits bandwidth. The web browsing behaves like the burst traffic model, i.e., a download uses 2 Mbps on average, while having “peaks” bursting up to 2.4 Mbps. Each downloading of a web consumes total bandwidth of 6 to 12 Mbits on average and the user will spend 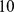 seconds on average to read the web.

To serve the two traffic types, a simple round-robin scheduler is built. Each request (video or web downloading) has at least a resource block quota for service when 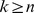. If 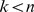, the scheduling policy among nodes will be based on the round-robin basis. The scheduler serves each video streaming request at most 2000 frames (10 seconds). If a video transmission has been served 10 seconds, the scheduler will release the channel and serve another request.

**Figure 10:**
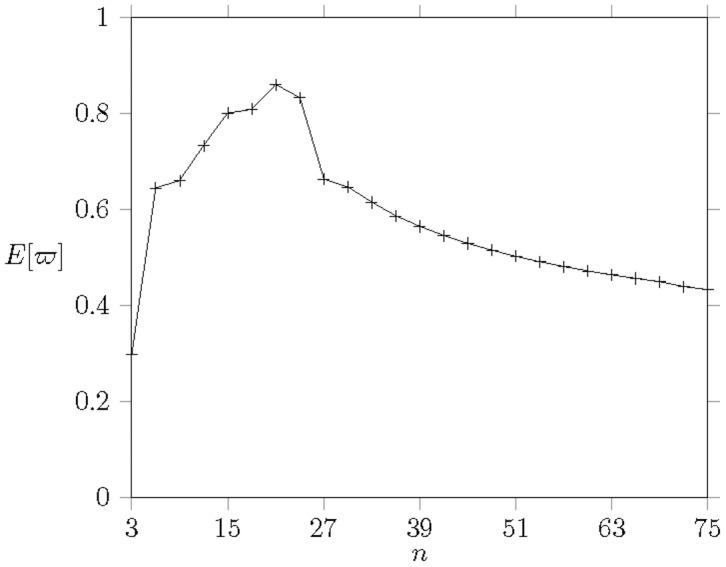
The energy consumption against the number of MSs 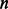 in realistic traffic where the ratio of video traffic to web traffic is 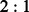.

The simulation results are shown in [Figure 10]. [Figure 10] shows the obtained results are very close to the numerical results. We notice that the network load is not saturated when 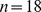 (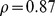) because there are 6 web requests (dominating 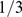 total traffic). As we mentioned that the web browsing behaves like brusty traffic. Therefore, the web browsing traffic only dominates one resource block a frame sustaining 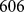 frames (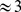 seconds) and then releases the channel. As we saw from [Figure 10], EPSS can reflect the network load and enforce the MHs entering the sleep mode to conserve energy if they cannot obtain the bandwidth immediately. The realistic traffic study shows EPSS has the capability of saving more energy when MHs face the saturated condition.

**Figure 11:**
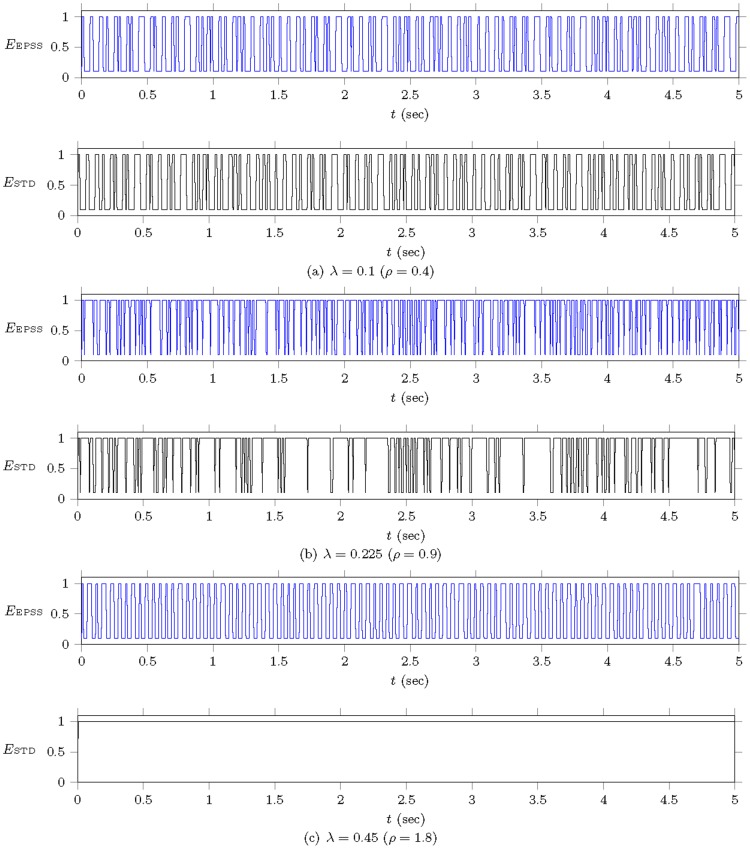
Example of energy consumption for EPSS and IEEE 802.16 in a single MH with 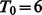, 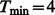, 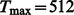, 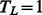, 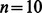, 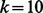, and 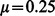. (a) middle traffic load 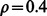. (b) saturated traffic load 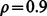. (c) overloaded traffic load 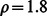.

[Figure 11] demonstrates the process of detailed energy consumption resulted by applying the EPSS and legacy IEEE 802.16 PSM. The simulation is performed for 5 seconds (1000 frames). To show the impact of EPSS on energy conservation, the simulation parameters are set as 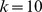, 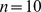, 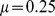, and 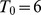. Three different traffic loads, 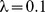 (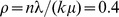) for a middle load, 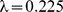 (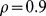) for a saturated load, and 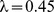 (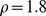) for an overloaded load, are investigated to observe the effectiveness of EPSS on energy conservation. The energy consumption in 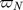 and 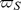 is 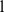 and 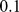. The energy consumption record is sampled at each frame. In the middle load ([Figure 11](a)), the energy consumption of EPSS, denoted by 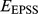, is similar with that achieved by IEEE 802.16, denoted by 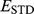. It is because that the traffic load is not saturated (i.e., 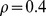) and the extended sleep mode is not activated. When the traffic load increases (i.e., 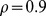 for saturated condition), as shown in [Figure 11](b), EPSS can enforce the MH to enter the extended mode for extra energy conservation as compared with the IEEE 802.16 PSM. Finally, we use an extreme case (i.e., 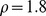 is sometimes taken by call admission control (CAC) for channel utilization improvement) to observe the effectiveness of EPSS. [Figure 11](c) shows that the MH adopts EPSS is able to enter the extended mode to conserve its energy when it cannot obtain the bandwidth in the active mode. This effect becomes more obviously when the traffic load is overloaded. For instance, visual recognition applications via mobile equipments for location identification are widely used recently [24]–[26]. These applications will query a great number of image and video and consume the bandwidth. In this case, EPSS has the capability of conserving the MH's energy. As compared with EPSS, MHs with the IEEE 802.16 PSM will wait for the downlink bandwidth and waste the energy all the time.

## Conclusion

In this paper, we invent a new power saving scheme, called *extra power saving scheme* (EPSS), to let MHs enter the *extended sleep mode* for extra power saving when the downlink traffic load is saturated. We show the impact of EPSS on energy consumption 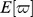 is significant when the number of MHs 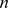 or the traffic load 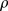 is large. We comprehensively show how the various parameters of the system, 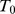, 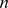, 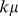, and 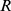, affect the performance of 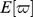. The performance analyses (i.e., the expected energy consumption and access delay) are verified by simulation, which includes an investigation of realistic traffic flows. Our invention gives a guideline to design PSM by considering the situation of downlink traffic load. EPSS can be adopted by the downlink scheduler of the BS to conserve the energy consumption of MHs. EPSS is suitable for applications that need a great deal of downloading such as mobile visual search. Moreover, how to combine with scheduling policies to optimize the efficiency of power saving is an important future work for study.
